# Death Attributed to Voudoo Incantations

**Published:** 1886-08

**Authors:** 


### *DEATH ATTRIBURED TO VOUDOO INCANTATIONS.*

A New Orleans coroner held an autopsy over the body of Henry Forschler, who died lately, the supposed victim of voudoo incantations and charms. The Forschlers are of German origin, but, like some white persons in Louisiana, believers in voudooism. When the young man was taken sick a regular doctor was called in to attend him, but he continued to grow worse. His father insisted that the sickness, which was of a mysterious character, was due to voudooism, and a mulatto, Glopion Grand, son of the famous voudoo queen, Marie Levan, and himself a practitioner of voudoo medicine, was called in. Glopion diagnosed the case and declared the young man had been charmed. Under his directions the floor was torn up, and a grigi, or voudoo charm, stuck full of needles was found buried there. He at once began his system of treatment, and the yonng man appeared on the road to recovery, when he suddenly passed away. The case had attracted a great deal of attention, and large crowds collected around the Forschler residence to see the man who had been voudooed. When his patient died Glopion was arrested, charged with murder, and locked up to await an autopsy. This was made, and showed that the death of Forschler was due to Bright's disease; but two-thirds of the negroes in New Orleans and some of the whites are convinced that the voudoo grigi did it. Glopion says he has been practicing voudoo medicine for over twenty years and numbers many whites as well as negroes among his patients. The matter has called attention to the large number of voudoo and quack doctors practicing among the negroes and poor whites of this city, and the board of health will try to break up their practice.—*Ex.*

